# A Genome-Wide Analysis of Array-Based Comparative Genomic Hybridization (CGH) Data to Detect Intra-Species Variations and Evolutionary Relationships

**DOI:** 10.1371/journal.pone.0007978

**Published:** 2009-11-24

**Authors:** Apratim Mitra, George Liu, Jiuzhou Song

**Affiliations:** 1 Department of Animal and Avian Sciences, University of Maryland, College Park, Maryland, United States of America; 2 Bovine Functional Genomics Lab, Animal and Natural Resources Institute, Agricultural Research Service, United States Department of Agriculture, Beltsville, Maryland, United States of America; Louisiana State University, United States of America

## Abstract

Array-based comparative genomics hybridization (aCGH) has gained prevalence as an effective technique for measuring structural variations in the genome. Copy-number variations (CNVs) form a large source of genomic structural variation, but it is not known whether phenotypic differences between intra-species groups, such as divergent human populations, or breeds of a domestic animal, can be attributed to CNVs. Several computational methods have been proposed to improve the detection of CNVs from array CGH data, but few population studies have used CGH data for identification of intra-species differences. In this paper we propose a novel method of genome-wide comparison and classification using CGH data that condenses whole genome information, aimed at quantification of intra-species variations and discovery of shared ancestry. Our strategy included smoothing CGH data using an appropriate denoising algorithm, extracting features via wavelets, quantifying the information via wavelet power spectrum and hierarchical clustering of the resultant profile. To evaluate the classification efficiency of our method, we used simulated data sets. We applied it to aCGH data from human and bovine individuals and showed that it successfully detects existing intra-specific variations with additional evolutionary implications.

## Introduction

Since the largest source of known genomic variations consists of single nucleotide polymorphisms (SNPs), an extensive amount of research has been conducted for characterizing SNPs in the human genome [1], [2]. A great volume of ongoing work has also involved finding the relationship between specific SNPs and human or animal disease. However, recent reviews [3] have suggested that structural variations, such as copy number variations (CNVs) and segmental duplications (SDs) may also be responsible, at least in part, for giving rise to complex disease. The advent of various high-throughput technologies such as array-based comparative genomics hybridization (CGH) has made it easier to probe such variations.

Array-based comparative genomic hybridization (aCGH) is becoming a popular and cost-effective way of detecting and measuring structural variations of the genome, and could be used for phylogenetic research. Although a wide range of computational methods exist [4], [5], [6], [7], the accurate estimation of copy number from CGH data is still an open problem. Segmentation approaches attempt to partition the genome into regions of ‘gain’ or ‘loss’, while denoising methods use distributional assumptions about experimental error to smooth the signal [5], [6], [8], [9], [10]. The latter are often coupled with a thresholding step to define regions of CNV [4], [7], [11]. There have been recent attempts to compare existing algorithms and to quantify their performance in detection of CNVs from aCGH [12], but no efforts have been directed towards using aCGH information for a population study. It is generally accepted that the ideal phylogenetic study should use genome-wide information from a large set of individuals, but this is impossible at present because of prohibitive cost, incomplete information and intensive computing requirements. Alternative ways are to use housekeeping genes, the largest possible DNA region or concatenation of core genes, which frequently result in biased inference and systematic overestimation within-species [13], [14], [15]. In addition, through decades of artificial selection and natural selection as well as population divergence created by geographical isolation, individuals from the same species have differences in their DNA sequences, which include SNPs and Structural Variations (SVs), although these differences cannot fully explain existing phenotypic differences. A recent effort to quantify the effect of genetic variation on gene expression found that SNPs and CNVs contribute 83.6% and 17.7% of the total variation found [16]. Therefore, the contribution of CNVs to genetic diversity is unquestionable [17].

In this paper, we propose a wavelet-based method to quantify structural variation profiles to enable comparisons between genomes of closely related individuals, which may pave the way for the use of genome-wide structural information for a phylogenetic study. We first use an appropriate denoising algorithmto smooth the CGH data and then extract features using wavelets. We quantify the information by calculating the wavelet power spectra, finally conducting hierarchical clustering of the resultant profiles to obtain a tree that serves to classify the individuals based on similarities in the CGH data. Our method is quite general; at every step we use well-known and often simple methods to achieve our aims. However, we are attempting to answer difficult questions: (a) Does CGH data contain adequate genetic information to enable us to distinguish between intra-species groups? (b) Are these patterns discernible over and above existing genetic diversity in a population?

Wavelet decomposition has been used for analyzing genome and gene expression profiles [18], [19], [20], [21], however, its application as a distance measure to find evolutionary relationships is novel. We validate the classification accuracy of our method using simulated data sets. Subsequently, we apply our method to human data sets from divergent populations [17] and bovine data sets from several breeds. These data sets capture varying degrees of genetic diversity in a natural population. We also conduct statistical tests to confirm the results of hierarchical clustering.

## Results

### Simulation Results

**Figure 1:**
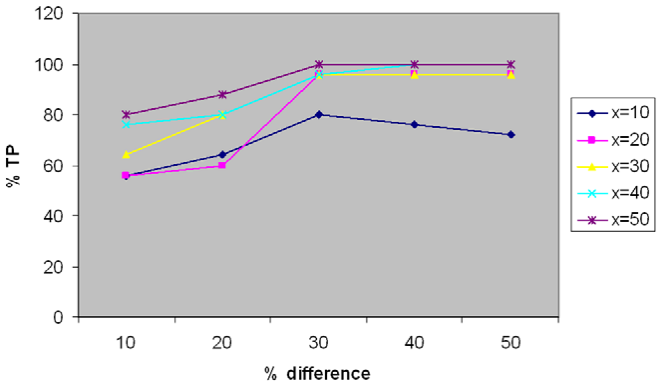
Plot of % TP vs % difference between groups, for changing values of total CNV number, x. The precision of the classification increases markedly with increasing divergence, with almost perfect classification for 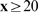, and % difference 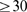.

To evaluate our method, the simulated data sets were generated with varying degrees of divergence between groups of individuals. The simulation parameters, *δ* and the total number of CNVs, *x*, were varied. For each experiment we simulated 25 individual genomes belonging to 5 different groups, each differing by δ*x CNVs. The true positives (TP) and false positives (FP) from the resulting hierarchical clustering tree were estimated by a simple rule: a cluster was assigned a particular group if more than 50% of its contents belonged to that group. The individuals that were correctly classified according to this rule were considered TPs. We plotted the % true positives from our simulation study. As shown in Figure 1, we found the precision of our classification increases with the extent of divergence, except when the total number of CNVs, *x*, is extremely low (*x* = 10). The accuracy is low when *x* is low, even when groups differ by as much as 50%. However, with increasing CNV, the FP rate decreases dramatically. For groups having a total of 40 CNVs (comparable to human CGH data sets), 24 out of 25 individuals (96%) were correctly classified for groups differing by 30%, with the number decreasing to 20 (80%) for groups differing by 20%.

Notably, the size of the CNVs in the simulation (1–10 probes), was much smaller than typical sizes considered for the validation of CNV detection algorithms (10–40 probes) in recently published studies [8], [12].

### Calculation of Hurst Parameter

The Hurst parameter, *H*, is a measure of self-similarity and *H* greater than 0.5 indicates the presence of long-range correlations. A recent study [11] has shown the presence of long-range correlations in aCGH data from a human individual suffering from cancer. Cancer is characterized by large scale CNVs and hence, such a finding is understandable. However, the data sets in our study are obtained from normal individuals and we establish the presence of long-range correlations to justify our use of the above de-noising algorithm.

**Figure 2:**
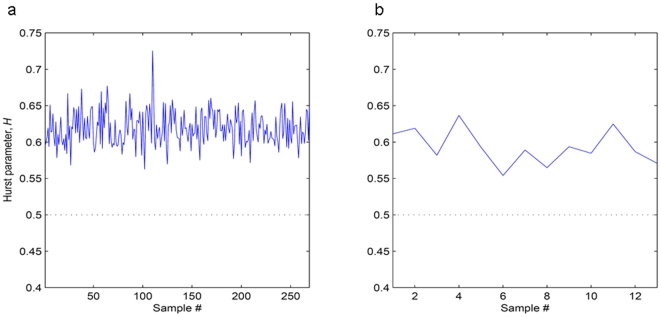
Plots of Hurst parameters from individuals. (a) 269 human individuals and (b) 13 bovine individuals. The dotted line corresponds to H = 0.5. The plots indicate the presence of long-range correlations in both human and bovine CGH data sets.

With the above objective in mind, we calculated the Hurst parameter for each of the human and bovine data sets as shown in Figure 2. The Hurst parameter, *H*, was found to be greater than 0.5 in all cases, confirming the presence of long-range correlations in our data sets. This proved the non-applicability of the normality assumption for aCGH data sets and hence, justified the use of our chosen aCGH de-noising algorithm.

### Human CGH Data Sets

The human CGH data sets for this study came from different populations and included individuals of African descent from Nigeria, European descent from Utah, USA, and Asian descent from China and Japan. We found some similarities between the Chinese and Japanese individuals and hence these samples were pooled together to form a third population alongside the African and European populations. We then carried out hierarchical clustering of the human CGH data profiles (Figure S1). The hierarchical clustering tree showed some interesting features, with several individuals from the same population clustered together in different regions.

In order to optimize the cluster size and total cluster number and also to further explore the reasons for the apparent misassortment, we cut the tree at height 12, creating 26 clusters with a median size of 9.5. We obtained the observed frequencies of the three populations from this clustering and conducted a permutation test to establish statistical significance as follows: The contents of all the clusters were pooled and shuffled. The reordered list was partitioned into sets with the same size distribution as our original clustering. The observed counts in our original clustering were compared with those obtained from the reordered list. This process was repeated 10^5^ times and the p-value of the comparison was calculated as the fraction of times the counts were lower in the observed clusters than in the random reordering.

**Figure 3:**
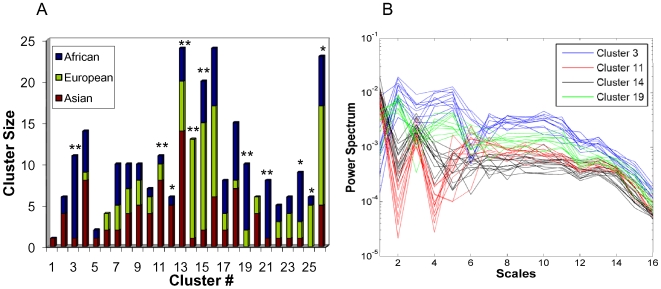
Plots of the clusters. (a) Bar graph showing relative proportions of the three populations in clusters obtained from the hierarchical clustering tree (Figure S1). Clusters are ordered by their location in the tree. Several clusters are highly statistically enriched for one population (p<0.01 ^**^) while some others are also moderately enriched (p<0.05^*^). (b) Plot of power spectrum profiles of highly enriched (p<0.01) clusters 3, 11, 14 and 19 showing distinct patterns. Cluster 3 and 19 consist of predominantly African individuals, cluster 14 consists of European individuals and cluster 19, Asian individuals.

The relative proportions of the three populations in the clusters are depicted in Figure 3(a). We found that 11 out of the 26 clusters (42.3%) were significantly enriched (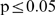) for one population, with 7 clusters (26.9%) highly enriched (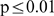). If we considered larger clusters (size 8 or larger), we got 16 clusters, covering 220 individuals (81.78%), nine of which were significantly enriched (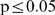). To demonstrate the presence of patterns in the power spectrum profiles, we plotted the spectra from individuals contained within certain clusters with high statistical enrichment, as shown in Figure 3(b). We also found some interesting clusters – cluster 4 and cluster 18 have similar proportions of Asian and African individuals, and clusters 7, 8, 9 and 16 have similar proportions of Asian, African and European individuals.

**Figure 4:**
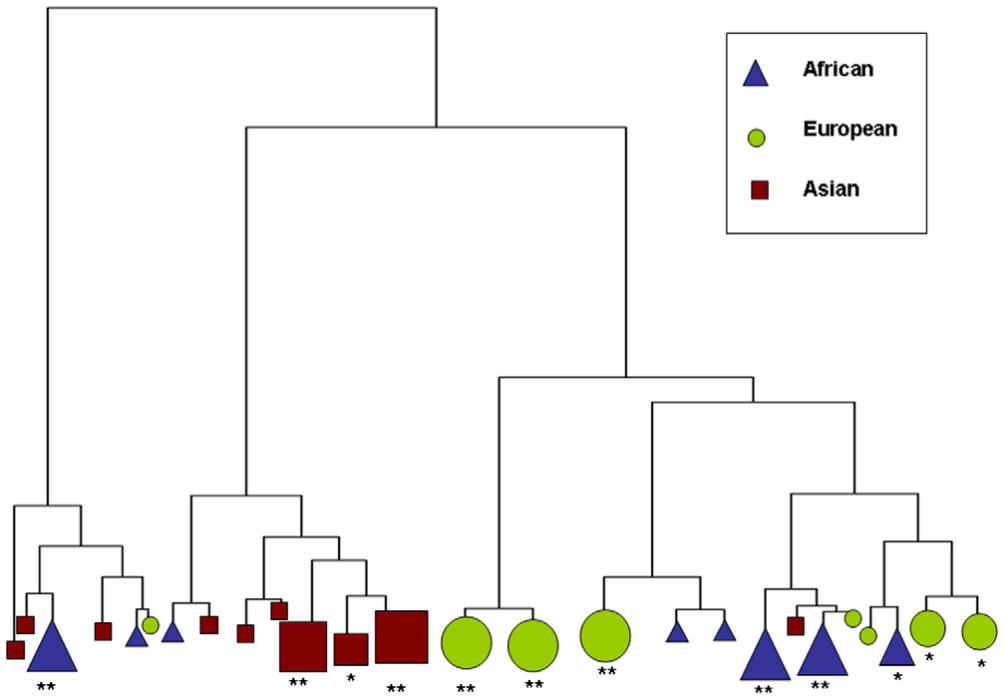
A hierarchical clustering tree showing the clusters colored by a majority rule. If more than 50% of the clusters contents belong to a particular population, the cluster is annotated according to this population. Size of the symbols indicate high statistical significance^**^ or moderate statistical significance^*^ as per the results of the permutation test.

On using a simple majority rule in deciding the annotation of a cluster (Asian, African or European) we get some interesting results (Figure 4). The tree has two major branches. The left branch consists mostly of Asian individuals, with only one cluster highly enriched for African individuals (p<0.01). These clusters form a small fraction of our total data set (14.13%), and could conceivable be outliers. The right branch consists of Asian individuals clustering at one end, European individuals clustering in the center and African individuals clustering at the other end. Hence, we see definite patterns of clustering, with individuals from the three populations clustering at distinct locations in the hierarchical clustering tree. We also see evidence that the African population has similarities with both Asian and European populations, while the latter two are more distant.

### Bovine CGH Data Sets

**Figure 5:**
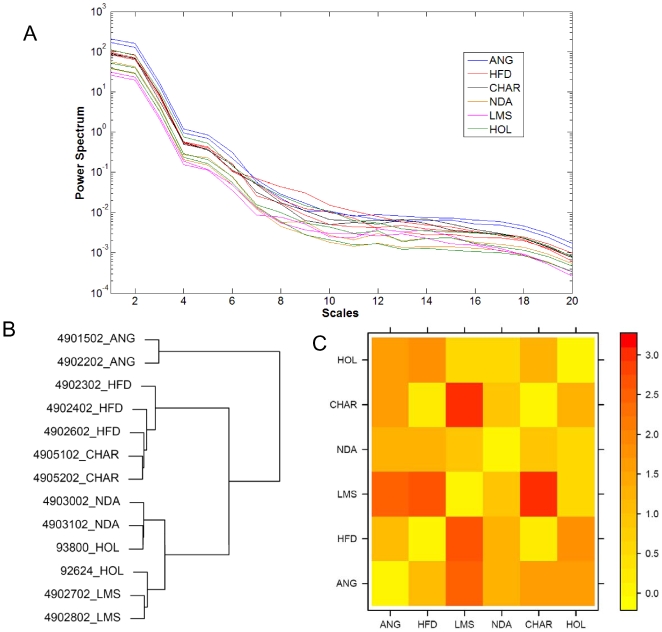
Plot of results of our method applied to bovine data sets. (a) Plot of power spectrum profiles and (b) Ward's hierarchical clustering tree for the 13 bovine individuals in our study and (c) Plot of –log_10_P values from comparisons of the breeds using the exact F-test (see Methods). Statistical significance (p<0.05) is indicated when –log_10_P = 1.3.

In an extended study, we analyzed the bovine CGH data sets generated in the Bovine Functional Genomics Laboratory, USDA. We had 13 individuals belonging to 6 different breeds: Angus (ANG), N'Dama (NDA), Holstein (HOL), Charolais (CHAR), Limousin (LMS) and Hereford (HFD). We expect a greater genetic variation between bovine breeds as compared to the human populations, with amplification of differences through several generations of artificial selection in cattle as opposed to imperfect geographical isolation for the humans. The power spectrum profiles from the bovine individuals were plotted (Figure 5(a)) and the hierarchical clustering of these profiles was carried out (Figure 5(b)). The plot of the spectra shows distinct patterns of similarity between individuals of the same species, a feature captured by the clustering tree. Besides, our test set in this case is much smaller than for the human data and hence, we manually curate the clustering tree. By cutting the clustering dendrogram at height 60, we obtained four well defined clusters. Considering a breed to be correctly classified if all the members of that breed are in a single cluster, we found that 5 out of the 6 bovine breeds and 11 out of the total 13 (84.6%) individuals in our data set are correctly classified. The other interesting aspect to note is the co-clustering of CHAR and HFD individuals.

We then used the exact F-test to check whether significant differences exist between breeds clustering separately [22]. The results of the statistical test confirmed the findings of the clustering tree and were plotted as a heatmap in Figure 5(c). We found the breeds that were in close proximity in the clustering tree, were not statistically different. For instance, NDA, LMS and HOL were not significantly different at the 5% significance level, but LMS is statistically different from ANG (p = 0.003), HFD (p = 0.0023) and CHAR (p = 0.00087).

The clustering tree therefore, provides some interesting findings. We found most of the individuals co-clustered with other individuals from the same breed with the exception of Holstein. The two HOL individuals are split between 2 clusters with NDA and LMS, but there is no significant difference between these breeds as shown by the results of the F-test. Moreover, 3 HFD individuals are co-clustered with CHAR individuals, and these breeds are also not significantly different.

## Discussion

In this study we have developed a new strategy towards using genome-wide information for a phylogenetic study and for classifying a population into distinct subgroups based on similarities and differences of aCGH data via wavelet spectra. Because wavelet analysis can capture the pattern information of a numerical signal by decomposition into time-frequency space, it has been used as a signal processing technique in many diverse fields, such as signal and image processing, numerical analysis and statistics. The basic idea of the method is to transform a sequential profile into several groups of coefficients, each group containing information about features of the profile at a different scale. In our study, the spectra represent structural features of the genome and reflect a compressed and comprehensive representation of structural variations consisting of SNPs, CNVs, SDs, etc. Moreover, it is executable on standard desktop computers and is not computationally demanding. This makes it easier to conduct comparative genomics and large-scale phylogenetic studies without being constrained by logistics. It is worthy of note that in our simulation the classification problem we consider is harder than the real case as the simulated CNVs contain fewer probe-sets and are shorter on average than CNVs from real aCGH data. Hence, the results are more remarkable.

In further studies, we analyzed data from two independent sources: 269 human CGH data sets from three populations and 13 bovine CGH data sets from 6 different breeds. In case of the human data sets, we analyzed the contents of the clusters using a permutation test and found 42.3% of the clusters to be significantly enriched for one population. The clustering tree showed groupings of Asian, European and African individuals in distinct regions. Moreover, African individuals seem to share similarities with both Asian and European individuals, while the latter appear to be less related. Our results are in accordance with the generally-held view that *Homo sapiens* originated in East Africa before spreading to different parts of the globe [23]. However, surprisingly we also found some clusters with comparable proportions of the three populations, indicating a broad base of similarity between them. Using our approach on the bovine data sets we were able to successfully classify 11 out of 13 individuals (84.6%) and 5 out of 6 breeds. The clustering tree uncovered putative evolutionary relationships between bovine breeds CHAR, HFD and NDA, LMS, HOL that were previously unknown. Our findings are supported by the results of an exact F-test which show that these sets of breeds are not significantly different amongst themselves.

The clustering trees from both species exhibit distinct characteristics. While the tree from bovine samples has clearly demarcated regions with most breeds clustering separately, the tree from human samples is more diffuse. This is consistent with the characteristics of the two populations: The human population possesses greater genetic diversity within populations as compared to between populations due to migration, mixing and imperfect geographical isolation. The low between-group variation in humans leads to a diffuse clustering tree with certain individuals from different populations appearing more similar than those from the same population. In comparison, the bovine population is quite the opposite, with higher variation between breeds due to artificial selection and conscientious isolation of genetic pools. The higher between-group variation makes it easier to distinguish between bovine breeds leading to a more clear-cut demarcation. This shows that our strategy is sensitive towards detecting underlying patterns of similarity within populations even in the presence of genetic diversity.

There are other possible reasons for the qualitative difference in the clustering trees from the two species: First, the de-noising algorithm used by us [11], although realistic, depends on a user-defined threshold *T* that can define performance. We conducted a systematic search of the parameter space to find the optimal value of *T* and reduce false positives. However, it is possible that the results will improve for a different value of the parameter. Notably, this is the only step of our method that requires choice of parameters. Alternatively, the use of a non-parametric smoothing algorithm such as (mean-shift-based) MSB [8] could produce an improved classification. Second, the array platforms for the two species have different resolutions with much higher resolution for the bovine data sets. In other words, the CGH data from humans may not detect small CNVs due to lower resolution and the differences that arise due to fine structural variations are therefore not picked up. The oligonucleotide arrays used for the cattle data sets do not suffer from this limitation and this may be one of the reasons behind the more diffuse clustering for the humans compared to the bovines. Third, we use the wavelet power spectrum to compress information from the wavelet decomposition by calculating sum-of-squares of the coefficients at each scale; the resultant profiles are subsequently clustered. This step is analogous to comparing energies of electrical signals with different scales corresponding to different bands of wavelength. The individual peaks of the wavelet decomposition may be different but the overall energy may be the same and consequently some differences may be missed. However, the fact that this strategy is very successful for the bovine data set shows that there is definite merit in this approach and a more involved scoring function based on the wavelet coefficients could produce marked improvements in classification. Lastly, the sample sizes in the two test sets are very different. The human CGH data set, although of a lower resolution, has a much larger number of individuals and consequently encapsulates much greater individual variation than the bovine data set. This coupled with the lower resolution makes it a difficult classification problem and could explain why we obtain a diffuse clustering tree.

Finally, no discussion related to copy number information can be completed without a mention of next-generation sequencing technologies and the alternatives they provide to aCGH. Genome-wide read counts provide a natural means of CNV detection without the limitations of intensity measurements that are characteristic of array-based techniques. In this study, we consider aCGH data as a discrete signal, and hence our approach is directly applicable to copy number data from such next-generation sequencing technologies.

In summary, our primary objective in this study was to verify whether CGH data representing genome-wide information can be used to distinguish between distinct groups or to do phylogenetic research via a wavelet-based novel strategy. Our results suggest that the idea is feasible. A logical next step in improving the power of inference would be to combine the CGH profiles with SNP information and investigate possible patterns of association between the occurrence of SNPs and CNVs. The advent of SNP-arrays have enabled recent efforts to combine SNP and CNV information in genome-wide association studies [24]. An extension of our method to this problem can potentially lead to interesting results.

## Methods

### CGH Data Sets

We use 269 human CGH data sets from a previous study [17] that used whole genome tiling path (WGTP) CGH arrays each having 26,574 probes. The samples are from four distinct populations: 90 individuals are of European descent, 45 from Japan, 45 from China and 89 from Nigeria and a sample from male individual of European descent served as the reference. We also analyze our bovine CGH data taken from 13 individuals from 6 different breeds, namely, Hereford (HFD), Charolais (CHAR), Limousin (LMS), N'Dama (NDA), Angus (ANG) and Holstein (HOL). The data is obtained from NimbleGen 385k oligonucleotide CGH arrays and the reference sample was blood taken from the sequenced Hereford cow. The study was specifically approved by USDA-ARS standard animal care procedures and guidelines.

Since our primary objective is to establish the fact that features extracted from CGH profiles can be used to classify different groups within the same species, the data was pre-processed to eliminate unwanted sources of variation or bias as far as possible. The reference sample for the human data sets was a male individual while that for the bovine data sets was female. However, the test samples from humans included both male and female individuals while the bovine data set consisted of male individuals and the data corresponding to the sex chromosomes may introduce biases into the study. Therefore, in order to remove sex-related effects, the data from the X chromosome was removed from both human and bovine data sets. We also removed the data from the unannotated and highly variable ChrUnAll region of the bovine genome.

### Simulated Data Sets

We created simulated data sets to validate the efficacy of our proposed method in distinguishing groups of closely related individuals based on their CGH profiles. We simulated artificial “genomes” of length 300,000 bp with CNVs uniformly distributed across the genome. Our strategy was to create a controlled difference between groups of individuals while keeping the rest of the genome relatively unchanged.

Assuming we have *m* groups of equal size *n*, we generated CNVs such that each group differs from the others by a fixed percentage. Keeping the number of CNVs in each individual at a constant level, say *x*, we define δ as the fraction of CNVs unique to each group as a measure of divergence, i.e. with increasing δ, the groups become more divergent. We kept within-group variation to a minimum to set up the baseline performance of our method.

For our simulations we chose *m = 5* and *n = 5* and varied δ from 0.1 to 0.5. The average number of CNVs detected per individual [17], was 35 for the WGTP arrays. Therefore, in our simulations, we increase *x* in steps of 10 from 10 to 50. The median size of the CNVs detected by the WGTP arrays was 228 kb, but this is partly due to the lower resolution offered by the large-insert clones of the platform and in case of oligo-based CGH arrays, the resolution is much higher (∼10 kb). In order to accommodate smaller CNVs we vary the size of the CNVs from 10 to 100 kb in our simulation.

The copy number states, *k*, of these segments are chosen to be between −6 to +6, based on the range of log-ratios observed in the bovine CGH data. We assume larger CNVs are less frequently observed, and the probability of choosing a particular copy number state *k* is proportional to 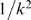. We add simulated noise to our genome from a Gaussian distribution of mean 0 and standard deviation 0.3, as the median standard deviation observed in our data sets was 0.26.

### Denoising Algorithm

There are several existing methods and algorithms for detection of regions of copy number change from noisy CGH data. However, a majority of approaches make normality assumptions about experimental noise. A recent study [11] has shown the presence of long-range spatial correlations in array CGH data and utilized this property to improve detection of regions of copy number change. Since we found the existence of long-range spatial correlations in our data, we use this algorithm for smoothing the CGH data in our study. Here is a brief overview of the procedure.

CGH data, *y*, of size *N*, is pre-processed using a median filter with a sliding window of length 3. A random walk, *y_RW_*, is constructed from the filtered data *y_f_*, by conducting a partial summation.
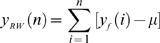
(1)where, 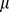 is the mean of *y_f_*, and *n* = *1,2, …* N. The remainder of the algorithm is based on the properties of a random walk. *y_RW_* is divided into overlapping windows within which local trends are calculated as the ordinates of linear least-squares fit for the random walk. The deviation between *y_RW_* and the local trend is denoted by *y_DEV_* that can be also be used to calculate the Hurst parameter. The original filtered data, *y_f_*, is then transformed in the following manner, to accomplish both segmentation and smoothing.
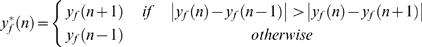
(2)


We define, 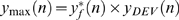. The smoothed data is given by,
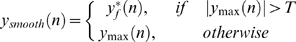
(3)where, T is a user-defined threshold. We use this smoothed data for further processing using wavelets.

We conducted an exhaustive search of the parameter space and found that this algorithm performs best with a threshold (T) of 0.5 for the human data sets and 0.75 for the bovine data sets. We found these values to be partly determined by the level of noise in the data. The human data had fewer data points and was less noisy as compared to the bovine data sets, and hence we needed a lower threshold for good performance. We anticipate the use of varying thresholds for different experiments for optimal results.

### Long-Range Spatial Correlations: The Hurst Parameter

CGH data from human or bovine individuals have not been previously shown to have spatial correlations. We test our data sets for the presence of spatial correlations by calculating the Hurst exponent [25], which can help detect patterns of self-similarity, for our dataset. We employ a simplified version of the classical Hurst's rescaled range algorithm for the calculation of the Hurst exponent.

Let us denote the CGH data of an individual by *y(n)* where 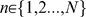. N denotes the number of data-points which, in this case, is the number of probes along the entire length of the genome. We define a vector 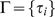 denoting the window size, where 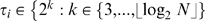 and divide the data *y* into 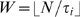 non-overlapping segments of size 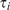. Let 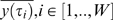 denote the mean of each such segment and the cumulative sum of deviations,
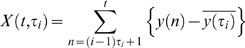
(4)


Where *t* = 1, 2, … *iτ_i_*. We, then, define *R* as,
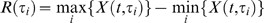
(5)and the standard deviation, *S*,
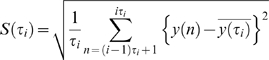
(6)


We calculate the mean of the rescaled range,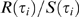, over all segments. The Hurst parameter, *H*, is given by the following relation,
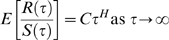



We plot 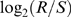 vs. 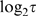 and the slope of the linear regression gives us an estimate of the Hurst parameter, *H*. The presence of long-range correlations is implied if 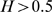.

### Feature Extraction with Wavelets

Wavelets have been used extensively in electrical engineering and biomedical research for signal processing and diagnostic purposes, respectively. They have been used as a tool for analyzing temporal data, such as, gene expression profiles [26], as also for the specific purpose of denoising CGH data by thresholding wavelet coefficients [4]. We propose to use wavelets for extracting and quantifying structural variations from the CGH data and classifying it into distinct levels or ‘scales’.

The discrete nature of CNVs and the lossless property of wavelet decomposition suggested their use in solving this problem. Since different wavelets produce the same or similar results, we use the Haar wavelet as the wavelet mother function for its simplicity and ease of use. It can be defined as,
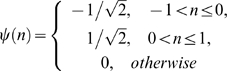
(7)


This wavelet is dilated and translated to form the wavelet basis according to the following relation:
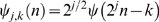
(8)where, *j* and *k* denote the scale of the decomposition and the position of the wavelet respectively. The data, *y(n)*, can then be represented as a linear combination of the wavelets as follows,
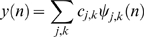
(9)where, 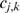 denotes the wavelet coefficients.

### Wavelet Power Spectrum

A natural way of quantification of the wavelet decomposition, is the wavelet power spectrum, which has previously been used for processing various biomedical signals, e.g., electrocardiogram (ECG), electroencephalogram (EEG) and heart rate variability (HRV) data, providing valuable diagnostic potential that can have far-reaching consequences in medicine [27], [28], [29] and also as a classification tool for gene expression profiles [30].

The wavelet decomposition divides the information from genome-wide CGH data into J scales. The wavelet power spectrum is calculated as follows:
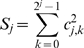
(10)where, 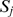 denotes the power spectrum at scale *j*, where *j* = 1, 2, … J, and 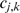 are the wavelet coefficients as in eq. (9). The power spectrum for each individual is calculated at several levels to generate a power spectrum profile which facilitates genome-wide comparison and classification using various clustering techniques.

We use cluster analysis in order to classify the power spectrum profiles after a log_2_ transformation. Various clustering strategies were tested including distance-based methods like average linkage and partition-based methods like *k*-means clustering. We finally choose Ward's method of hierarchical clustering [31] that merges clusters resulting in the smallest increase in information loss, defined in terms of an error sum-of-squares criterion.

### Statistical Analysis

We evaluate the statistical significance of differences between power spectrum profiles, by a transformation of *q*-fold principal components (PC) [22], [32].

Let 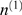 and 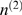 represent the number of individuals in two groups to be compared, such that, 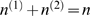. If we have the wavelet power spectrum for each individual at *p* scales, we define 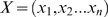 as a 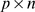 matrix representing the power spectrum values. Assuming 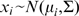, the null hypothesis to be tested is,
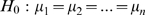
(11)


Let us denote 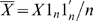, and let *D* be a 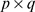 matrix consisting of the first *q* 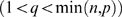 eigenvectors of the solution to the following general eigenvalue problem,
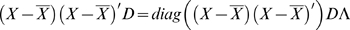
(12)where 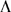 is the 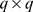 diagonal matrix of the *q* largest eigenvalues.

If *H_0_* holds, the statistic,
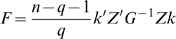
(13)where 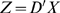 and 
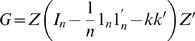
, exactly follows the *F* distribution with *q* and *n-q-1* degrees of freedom. *k* is a vector calculated according to following equation
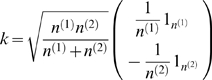
(14)


For given *n* and *p*, the power of this statistic depends on *q*, the number of principal components to be considered. We determined this parameter based on a cumulative energy content (CEC) criterion, such that, the *q* principals capture at least 85% variance in the data.

## Supporting Information

Figure S1 Figure S1
(0.12 MB EPS)
